# Fast Bragg Grating Inscription in PMMA Polymer Optical Fibres: Impact of Thermal Pre-Treatment of Preforms

**DOI:** 10.3390/s17040891

**Published:** 2017-04-18

**Authors:** Carlos A. F. Marques, Andreas Pospori, Gökhan Demirci, Onur Çetinkaya, Barbara Gawdzik, Paulo Antunes, Ole Bang, Pawel Mergo, Paulo André, David J. Webb

**Affiliations:** 1 Instituto de Telecomunicações and Physics Department & I3N, Universidade de Aveiro, Campus Universitário de Santiago, 3810-193 Aveiro, Portugal; pantunes@av.it.pt; 2 Aston Institute of Photonic Technologies, Aston University, Aston Triangle, B4 7ET Birmingham, UK; posporis2@gmail.com (A.P.); d.j.webb@aston.ac.uk (D.J.W.); 3 Department of Polymer Chemistry, Maria Curie-Sklodowska University, 20-031 Lublin, Poland; luapy2004@gmail.com (G.D.); barbara.gawdzik@poczta.umcs.lublin.pl (B.G.); 4 Laboratory of Optical Fibre Technology, Maria Curie-Sklodowska University, 20-031 Lublin, Poland; onrctnkya@hotmail.com (O.Ç.); Pawel.Mergo@umcs.lublin.pl (P.M.); 5 Department of Photonics Engineering, Technical University of Denmark, DK-2800 Kongens Lyngby, Denmark; oban@fotonik.dtu.dk; 6 Instituto de Telecomunicações and Department of Electrical and Computer Engineering, Instituto Superior Técnico, University of Lisbon, 1049-001 Lisbon, Portugal; paulo.andre@lx.it.pt

**Keywords:** polymer optical fiber, annealing process, Bragg gratings, preform treatment, POF sensors

## Abstract

In this work, fibre Bragg gratings (FBGs) were inscribed in two different undoped poly- (methyl methacrylate) (PMMA) polymer optical fibres (POFs) using different types of UV lasers and their inscription times, temperature and strain sensitivities are investigated. The POF Bragg gratings (POFBGs) were inscribed using two UV lasers: a continuous UV HeCd @325 nm laser and a pulsed UV KrF @248 nm laser. Two PMMA POFs are used in which the primary and secondary preforms (during the two-step drawing process) have a different thermal treatment. The PMMA POFs drawn in which the primary or secondary preform is not specifically pre-treated need longer inscription time than the fibres drawn where both preforms have been pre-annealed at 80 °C for 2 weeks. Using both UV lasers, for the latter fibre much less inscription time is needed compared to another homemade POF. The properties of a POF fabricated with both preforms thermally well annealed are different from those in which just one preform step process is thermally treated, with the first POFs being much less sensitive to thermal treatment. The influence of annealing on the strain and temperature sensitivities of the fibres prior to FBG inscription is also discussed, where it is observed that the fibre produced from a two-step drawing process with well-defined pre-annealing of both preforms did not produce any significant difference in sensitivity. The results indicate the impact of preform thermal pre-treatment before the PMMA POFs drawing, which can be an essential characteristic in the view of developing POF sensors technology.

## 1. Introduction

POFs can be considered as a strong alternative to silica fibres, in applications such as short distance optical links, Terahertz waveguides and filters, and mainly in sensing applications [1,2,3,4] due to their flexibility, high failure strain, large cores and great elasticity. The mechanical properties provide enhanced sensitivity or longer operational range to intrinsic polymer fibre sensors when they are used for strain, stress, pressure, temperature and humidity monitoring, as well as for transverse force sensing [5,6,7,8,9,10,11,12]. Many of these sensors are based on FBGs, which have been written in different spectral regions in doped and undoped step-index POFs [13], microstructured POF (mPOF) (including PMMA and TOPAS materials) [13,14,15], as well as low loss cyclic optical polymer (CYTOP)-perfluorinated POFs [16], and graded-index POFs [17]. Polymer optical fibre Bragg gratings (POFBGs) can be inscribed with different laser systems including continuous-wave (CW) HeCd laser (@325 nm) [13,14,15], pulsed KrF laser (@248 nm) [18], and also femtosecond laser systems [16].

Fabrication of Bragg gratings in mPOF and step-index fibres, with the phase mask technique is a time consuming process. Using a 325 nm UV laser, in undoped mPOFs exposure times from 60 to 270 min have been reported [19,20], with the lowest inscription time reported being approximately 7 min [21]. For the step-index fibres the inscription times are shorter and typically around to 45 to 100 min [19,22] with the lowest inscription time reported being approximately 20 min [13]. The writing times can be reduced by doping the fibre [14], however doped fibres are more difficult and expensive to fabricate, the transmission loss increases and they are less suitable for in vivo biosensing. In 2015, in a way to help to manage it without the fibre doping, a 248 nm UV laser was used to inscribe Bragg gratings in undoped mPOF, at low fluence and low repetition rate (I = 33 mJ/cm^2^; R = 1 Hz) in a short time of around 30 s [18], showing that Bragg grating systems designed for silica fibres can be used to inscribe POFBGs, potentially increasing their take-up in more R&D laboratories. Also, very recently, chirped POFBGs were photo-inscribed after 14 pulses in an undoped POF [23].

In order to understand the fabrication process needed to achieve undoped POFs with good performance as well as to reduce the FBG inscription time, we compare different undoped PMMA POFs using two different UV lasers: a continuous UV HeCd @325 nm laser and a pulsed UV KrF @248 nm laser.

In this paper, we provide evidence that a specific thermal pre-treatment of both preforms can be responsible for a better photosensitivity mechanism of undoped PMMA POF based sensors irradiated with UV light. In the experiments we observed that there is an increase of the material’s photosensitivity in samples subjected to a well-defined thermal pre-treatment of both preforms before the PMMA POFs drawing. 

## 2. mPOFs under Investigation and FBG Inscription Systems

**Figure 1:**
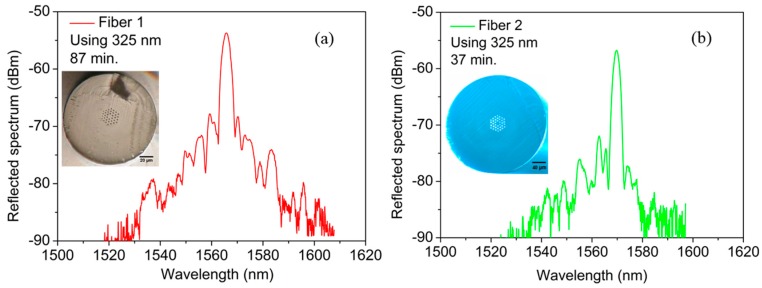
Reflected spectra for POFBGs inscribed in (**a**) Fibre 1 and (**b**) Fibre 2 using the CW 325 nm UV HeCd laser. Insets: Cross-section images of the POFs used in this work.

Two different undoped PMMA mPOFs, labeled Fibre 1 and Fibre 2, were drawn in different facilities, where Fibre 1 is an mPOF referenced in [24] and Fibre 2 was fabricated at the Maria Curie-Sklodowska University, Lublin, Poland, being also an mPOF. The fibre core and cladding diameters of the fibres are, respectively, 8/135 μm (Fibre 1) and 9/270 μm (Fibre 2). The core of the fibres is composed of poly-methyl methacrylate (PMMA) with no additional dopants, whilst the cladding is also made of PMMA. Both fibres have a three-ring hexagonal cladding structure, and were fabricated from commercial PMMA material. However, we are under the assumption that the amount of unpolymerized monomers and the storage conditions, as well as the preform drilling are quite similar between the manufacturers. Typically, the drawing process can be done in two-step process where first we draw a cane, then sleeve it, and finally we draw the secondary preform. Both fibres were drawn in this way. However, the preform of Fibre 2 was pre-annealed during both preform two-step drawing process—primary and secondary preforms—for 2 weeks at 80 °C in each step before the fibre was drawn. For Fibre 1, the preform was not annealed in the same way, since only the secondary preform was annealed—at 80 °C during a week. Important parameters of the fibres used (hole diameter/pitch, draw ratio, pulling speed, drawing temperature and tension) are summarized in Table 1. The cross-section images of the POFs used in this work are shown in the inset of Figure 1.

Two different inscription systems were used to inscribe FBGs in order to compare their performance in each fibre. The first system is based on a 325 nm UV light from a CW HeCd laser (KIMMON laser systems, Centennial, CO, USA) with a power output of 30 mW and a beam diameter of 1.2 mm [8]. The HeCd laser beam was focused vertically downward (using a 10 cm focal length cylindrical lens) through the phase mask designed for 325 nm operation, and onto the fibre. POF sections 10 cm long were laid in a v-groove and taped down using polyimide tape to prevent them from moving during inscription. With this system, the inscription process was monitored using a broadband light source (provided by an ASE-FL7002-C4, Thorlabs, Newton, NJ, USA), and an optical spectrum analyzer connected to an optical coupler. The second system is based on a pulsed KrF Bragg Star^TM^ Industrial-LN excimer laser (Santa Clara, CA, USA) operating at 248 nm [18]. The laser has a rectangular Tophat beam spot of 6 mm width and 1.5 mm height, with pulse duration of 15 ns. A cylindrical lens, followed by a slit with 4.5 mm width, shapes the beam before it arrives to the phase mask, designed for 248 nm operation. 18 cm long POF sections were placed within two magnetic clamps and kept in strain to avoid undesired curvatures. Here, an interrogation system (sm125, Micron Optics, Atlanta, GA, USA) was used to monitor the grating growth.

In all cases, POF sections were cleaved with a hot blade on a hot plate (at 70 °C) and then a butt-coupled connection was made between one arm of a single-mode silica coupler and the POF using an FC/APC connector on the silica fibre. A small amount of index matching gel was used in order to reduce Fresnel reflections, lowering the background noise. To compare the FBG reflected amplitude, all the FBGs used in this work were inscribed at the same distance from the FBG monitoring input. The butt-connection loss was minimized by optimizing the alignment between the two fibre types using a 3D micrometric translation stage. This was controlled using a power measurement in transmission as well as the noise level in the measured reflected spectrum.

## 3. Results and Discussion

Several FBGs in each pristine fibre sample were produced using both FBG inscription systems. Figure 1 shows the reflected spectra for the two POFs using the 325 nm UV HeCd laser. The inscription times (the time in which the grating growth stops) for Fibres 1 and 2 are on average 87 min and 37 min, respectively, after several inscriptions to make sure about the repeatability of the results. We can notice that for the latter fibre less than half the inscription time is needed compared with Fibre 1. We shall recall that the preform from each drawing process of Fibre 2 has been annealed for 2 weeks at 80 °C, giving a well-defined thermal pre-treatment when compared with Fibre 1. The effect of annealing on a POF in which the preform has been annealed prior to drawing is different as reported and discussed recently in [25], where this fibre type is far less sensitive to thermal treatment.

**Figure 2:**
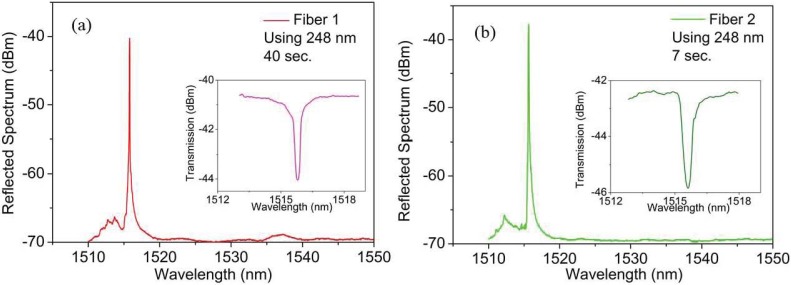
Reflected (inset: transmission spectrum) spectra for POFBGs inscribed in (**a**) Fibre 1 and (**b**) Fibre 2 using the pulsed 248 nm UV KrF laser.

To substantiate our findings, we repeated the same measurements on the two fibre samples but now using the 248 nm UV KrF laser. The laser parameters were set to a frequency of 1 Hz and a pulse energy of 3 mJ. Figure 2 shows the reflection and transmission spectra and for this case the inscription times for Fibres 1 and 2 were on average 40 s and 7 s, respectively. For Fibre 2, the optimum irradiation time was estimated to be 7 s meaning that only 7 pulses where needed to produce a saturated refractive index change. In Fibre 2, where the both preforms had been annealed prior to drawing, the inscription time is also lower than the inscription time needed for the fibre sample 1 (in fact, we need 5 times less of the total inscription time using Fibre 2), meaning that this is the same situation as for the case with inscription using the 325 nm UV HeCd laser. Table 2 summarizes the central wavelength, full width at half maximum (FWHM) and reflectivity of each FBG inscribed for both inscription systems. Note that we have used different FBG lengths in each inscription system: 1.2 mm and 4.5 mm FBG lengths using 325 nm and 248 nm laser, respectively, hence we obtained different bandwidths.

As it is well known, the angular orientation of the fibre microstructure has a substantial influence on the intensity distribution of the UV beam in the core section, which affects the growth dynamics [21]. However, in our case, the success rate of the photo-inscription is quite high—more than 95%.

**Figure 3:**
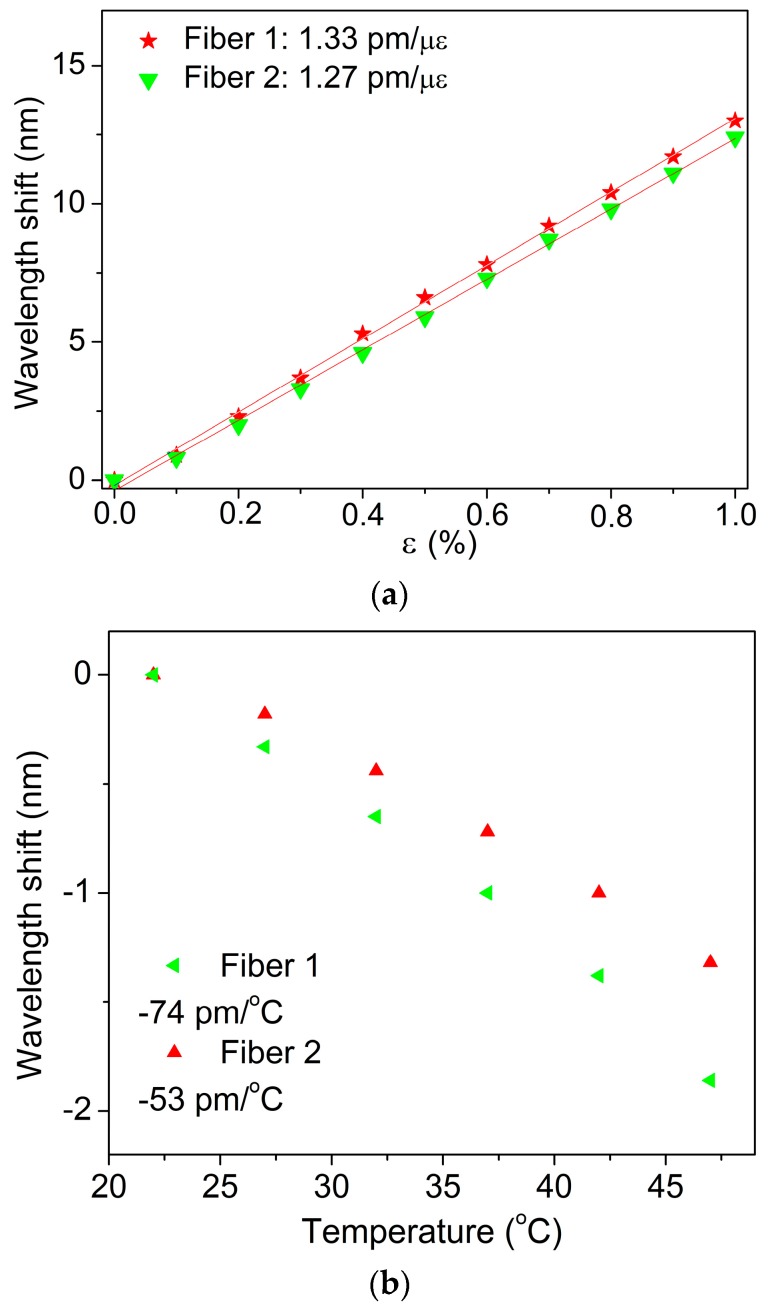
Bragg wavelength shifts obtained from the inscribed FBGs in each fibre under different (**a**) strains and (**b**) temperatures.

The performance of the produced sensors in terms of strain and temperature sensitivities was analyzed. A strain characterization was performed in order to show the spectral dependence of the Bragg reflection peak with the strain for each fibre using FBGs inscribed by both laser systems. The results are shown in Figure 3 and as it can be seen the Bragg wavelength shift was linearly red shifted with 1% deformation. The obtained strain sensitivities for FBGs inscribed were 1.33 ± 0.01 pm/με (Fibre 1) and 1.27 ± 0.02 pm/με (Fibre 2) after using a linear regression fit, where the results are similar to the typical values already reported in literature for POFBGs (~1.3 pm/με in the 1550 nm window) using both UV laser inscription systems [17,18].

Additionally, characterization was carried out to explore the temperature response of each fibre containing FBGs. The fibres were placed in an environmental chamber under varying temperatures to study their response. The temperature was increased from 22 °C up to 47 °C with steps of 5 °C. In each step, the temperature was kept constant over 35 min to ensure that thermal equilibrium was achieved. The temperature characterization was done at a fixed 50% relative humidity. The obtained temperature sensitivities were similar to the values already reported for POFBGs inscribed in Fibre 1 with 325 nm laser system [26]: −74 ± 3.3 pm/°C. For Fibre 2, we achieved a temperature sensitivity of −53.1 ± 2.2 pm/°C, which is less than the value achieved for Fibre 1, as discussed in [25], suggesting that these POFs are much less sensitive to thermal treatment due to the impact of preform thermal pre-treatment before the PMMA POFs drawing.

The results presented here indicate the impact of thermal pre-treatment in both preforms processes before the PMMA POFs drawing on the fast inscription of POFBGs, which is an essential characteristic in the view of developing stable POFBG based thermo-mechanical sensors. The different drawing parameters, namely drawing tension from which we calculate the draw stress and the atmosphere in which the preform is placed during annealing, can also give us a credible explanation. However, using three fibres fabricated at different drawing tensions (from the same facility of Fibre 2) some FBGs were produced and no significant difference was observed in terms of inscription time between all fibres. Any annealing process prevents humidity to be diffused and consequently avoiding some issues during drawing such as bubbling, destabilized conditions or fluctuation on diameter of fibre. With this long time of annealing, all water quantity may be removed. So, if there is a fibre drawn from a preform which had not a specific process of annealing in any of two-step process (primary or secondary preforms), it could include a considerable amount of water inside the preform and can affects the final performance of the fibre. However, it does not mean that the final fibre in both cases will have a large different content of water at the end but a slight difference may be sufficient. For both fibres, the humidity of the oven is controlled by argon flow or in vacuum to guaranty that the atmosphere where the fibre is annealed is as free as possible from external climatic changes.

On the other hand, after inscribe FBGs on the pristine samples using both laser systems and discuss the obtained results above, in order to explore and understand our findings we then annealed new samples for 12 h at 80 °C before the FBGs inscription. PMMA based POFs are well known to be sensitive to humidity [27]. Whilst we could not control the history in terms of exposure to environmental temperature and humidity changes between their fabrication and arrival at our laboratories, we emphasize that in between the annealing steps carried out in our labs, all fibre samples were stored and measured in the same temperature (20 °C) and relative humidity (50%) controlled cleanroom and therefore in the same environmental conditions. Thus, we observed that the inscription times for Fibres 1 and 2 are on average similar to the previous case, meaning that there are no significant improvements regarding the inscription time consumption compared with non-annealed fibres before FBG inscription. 

Also, we extended our experiments to strain and temperature using the same procedure presented before. Table 3 summarizes the results obtained from the inscribed FBGs in each fibre under different strains and temperatures. There was a significant improvement around 5% for Fibre 1, suggesting that much higher strain can be applied to the annealed POFBG sensors [28]. For Fibre 2 we achieved no significant difference compared with non-annealed fibre before FBG inscription. The temperature sensitivities obtained for POFBGs inscribed in Fibres 1 and 2 are also presented in Table 3. As reported in [28], we achieved similar thermal sensitivities for all gratings (pristine and annealed fibres before FBG inscription), which means that the annealing process of fibres before FBG inscription does not change significantly the thermo-optic coefficient of the material. With this, we noticed that the strain and temperature sensitivities of Fibre 2 are not at all affected by the fibre annealing process before FBG inscription. Recall that the preform of this fibre has been annealed for 2 weeks at 80 °C in both two-step process and as reported in [25], this well-defined annealing of the preform plays a key role for the development of stable POFBG sensors, improving at the same time the inscription time of FBGs. 

## 4. Conclusions

In this work, improvements were reported in the photosensitivity of undoped POFs, where there was a well-defined pre-annealing of both the preforms fabricated in two-step process. We have observed that with non-annealed preform in any step of the process (in this case primary preform), the fibre photosensitivity is lower. The fibres from preforms with specific thermal pre-treatment in both two-step process allow us to achieve less FBG inscription times comparing to fibres with a well-defined annealing of the secondary preform, obtaining at the same time stable FBG sensors with high quality. We also addressed the actual influence of annealing on the strain and temperature sensitivities of the fibres prior FBG inscription, observing that the fibre produced with well-defined pre-annealing of both preforms did not produce any significant difference. Some important parameters were considered such as drawing tension where using fibres drawn in different tensions give us a similar FBG inscription time. We can also conclude that a fibre drawn from a two-step process, where at least the primary or secondary preforms is not annealed, may include a slight amount of water inside of the preform and this will affect the fibre’s performance. Also, the conclusions are under the assumption that the amount of unpolymerized monomers and the storage conditions, as well as the preform drilling are quite similar between the manufacturers.

## Figures and Tables

**Table 1:** Fibre parameters.

	Core/Cladding Size (μm)	Cladding Structure	Hole Diameter/Pitch (μm)	Draw Ratio (mm)	Pulling Speed (m/min)	Drawing Temperature (°C)/Tension (N)	Both Preforms Annealed?
Fibre Name							
Fibre 1	8/135	Three-Ring Hexagonal	1.9/4.3	20/0.135	40	290/0.20	No
Fibre 2	9/270		2.0/4.6	11/0.270	30	290/0.5–1.0	Yes

**Table 2:** Central wavelength, bandwidth and reflectivity of each FBG inscribed for both inscription systems.

		Using 325 nm Laser			Using 248 nm Laser	
Fibre Number	Central Wavelength (nm)	Amplitude (dB)	FWHM (nm)	Central Wavelength (nm)	Amplitude (dB)	FWHM (nm)
Fibre 1	1565.91	32.10	5.73	1515.80	29.88	0.16
Fibre 2	1569.82	31.58	5.21	1515.63	31.68	0.25

**Table 3:** Strain and temperature sensitivities for each POFBG using pristine fibre samples and pre-annealed fibres before FBG inscription.

	Strain Sensitivity (pm/με)		Temperature Sensitivity (pm/°C)	
Fibre Number	Pristine Sample	Annealed Sample	Pristine Sample	Annealed Sample
Fibre 1	1.33	1.40	−74	−75
Fibre 2	1.27	1.26	−53	−53
